# Efficacy and mechanism of acupuncture for functional constipation in older adults: study protocol for a randomized controlled trial

**DOI:** 10.3389/fneur.2024.1341861

**Published:** 2024-04-15

**Authors:** Yisheng Huai, Qian Fan, Yiyue Dong, Xu Li, Junwei Hu, Lumin Liu, Yuelai Chen, Ping Yin

**Affiliations:** Sleep Medicine Center, LongHua Hospital Shanghai University of Traditional Chinese Medicine, Shanghai, China

**Keywords:** functional constipation, acupuncture, randomized controlled trial, older adults, intestinal microbiota, inflammatory cytokines

## Abstract

**Introduction:**

Functional constipation (FC) is a common functional gastrointestinal disorder in clinical practice, with the prevalence of which increasing with age. With the increasing aging of the population worldwide, this problem is bound to become more prominent. Acupuncture is effective and recommended for the treatment of FC. However, little is known about how acupuncture affects the gut microbiota and inflammatory cytokines and thus improves gut function. Meanwhile, there are few high-quality clinical trials specifically focusing on acupuncture in treating FC in older people. The objective of this study is to assess the efficacy and safety of acupuncture in treating FC in older people. Additionally, the research aims to explore the mechanism of action of acupuncture in treating FC in older people by affecting intestinal microbiota and inflammation cytokines.

**Methods and analysis:**

This study is designed as a single-center, randomized, sham-controlled clinical trial. A total of 98 eligible FC patients will be randomized in a 1:1 ratio into an acupuncture group and a sham acupuncture group. Both groups will receive 24 treatments over 8 weeks with a 12-week follow-up. The primary outcome of the study is the treatment response rate, which is the proportion of participants with ≥3 mean weekly Complete Spontaneous Bowel Movements (CSBMs) over weeks 3–8. The secondary outcomes will include the proportion of participants with ≥3 mean weekly CSBMs during other assessment periods; the percentage of patients with ≥1 increase in mean weekly CSBMs from baseline; the average changes in CSBMs; Patient Assessment of Constipation-Symptoms (PAC-SYM), Bristol Stool Scale, Patient Assessment of Constipation Quality of Life Questionnaire (PAC-QOL), Self-rating Anxiety Scale (SAS), Self-rating Depression Scale (SDS) and weekly usage of emergency bowel medications. Adverse events will be recorded throughout the study. Data for the outcomes will be collected at Week 0 (baseline), Week 4 (the intervention period), Week 8 (the post-treatment), Week 12 (the follow-up period) and Week 20 (the follow-up period). In addition, changes in intestinal microbiota will be analyzed using 16S rRNA high-throughput detection, and the concentration of relevant inflammatory cytokines in serum will be measured by ELISA based on blood samples. The intention-to-treat analysis will be performed in this study.

**Clinical trial registration:** [https://www.chictr.org.cn/], identifier [ChiCTR2300070735].

## 1 Introduction

Functional constipation (FC) has attracted widespread attention due to its high prevalence in the elderly population. Its incidence gradually increases with age (1, 2). 18.1–33.5% of people over 60 years old develop FC (3, 4). Furthermore, people over 80 years old have a higher risk (5). The diagnosis of FC is based on the Rome IV criteria (6). However, the etiology of constipation is usually multifactorial with aging, lack of physical activity, inadequate water and dietary fiber intake, dysbiosis, substance use, and so on (7–10). Long-term constipation will not only lead to colon diverticulum, perianal disease, colon melena, and other anorectal diseases (11), but also become a common predisposing factor for cardiovascular and cerebrovascular diseases such as myocardial infarction and cerebrovascular accident (12, 13). Some patients with severe constipation can also suffer from psychological disorders such as insomnia, irritability, depression, and obsessive-compulsive thoughts and behaviors (14–16). Therefore, as the aging of the population increases, this disease will seriously affect patients’ quality of life and bring a heavier burden to society.

Currently, the treatments of FC include lifestyle modification, transanal irrigation, pharmacological interventions, biofeedback therapy, and probiotics (17–21). Among these treatments, nonpharmacological management is the first step in the treatment of FC. When lifestyle modification and dietary interventions are ineffective, patients will seek help from medications. However, medication-assisted treatment is always limited in older patients (22). This is because most older adults have chronic diseases that require long-term treatment with other medications. Considering that drug interactions may further aggravate the disease, it is imperative to find a therapeutic method with certain efficacy and few side effects. It’s worth pointing out that acupuncture has been suggested to show potential for the treatment of FC. Recently, acupuncture has been widely used in the treatment of FC with relatively satisfactory results (23, 24). These results of the studies provide evidence for the efficacy of acupuncture in the treatment of FC. Nevertheless, the mechanisms underlying acupuncture in the treatment of FC are still incompletely fully understood. In addition, there are no reports of high-quality clinical studies on acupuncture for the treatment of FC in older people.

In recent years, many studies have focused on intestinal flora’s effects on human health, disease, and aging (25–27). It is noteworthy that dysbiosis of the intestinal microbiota is associated with constipation. It is reported that microbial diversity and abundance often decline in patients with constipation (28). In addition, the abundance of beneficial bacteria in the gut decreases with age (29). The gut microbiota of older people is less diverse compared to that of young people (30, 31). Meanwhile, any disturbance in the composition of the microbiota may lead to a reduction in the efficacy of the immune system, which is more pronounced in the elderly. In addition, cytokines play an important role in regulating the immune system’s interactions with the microbiota. As humans age, the function of innate and acquired immune both decreases, and pro-inflammatory mediators, such as acute-phase proteins, cytokines, and adhesion molecules in the bloodstream increase (32).

The selection and combination of acupuncture points, which is a key factor in the clinical effectiveness of acupuncture, is also a characteristic of acupuncture treatment. Meanwhile, we have observed in our clinic that some patients with FC have not only improved their constipation symptoms but also their moods after receiving acupuncture treatment. Consequently, considering this and the efficacy of the combination of acupuncture points and pathological characteristics of FC in older people, we designed this study. We hypothesize that acupuncture might improve clinical symptoms in older patients with FC by modulating intestinal microbial composition and inflammatory cytokines. Consequently, this trial aims to assess the efficacy and safety of acupuncture in treating FC in older people and to explore these mechanisms.

## 2 Methods and analysis

### 2.1 Study design

**Figure 1:**
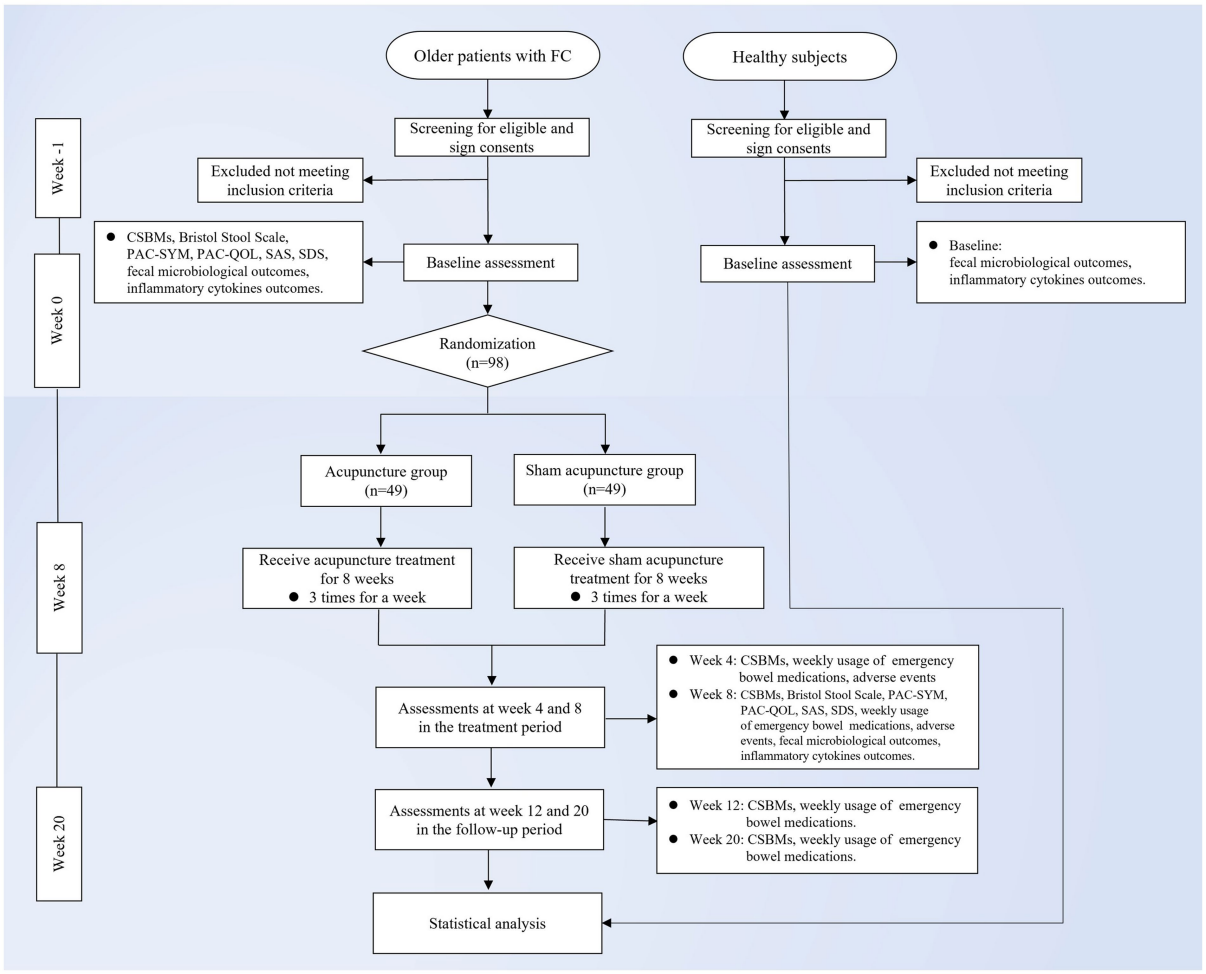
Trial flow chart. FC, functional constipation; CSBMs, Complete Spontaneous Bowel Movement; PAC-SYM, Patient Assessment of Constipation-Symptoms; PAC-QOL, Patient Assessment of Constipation Quality of Life Questionnaire; SAS, Self-Rating Anxiety Scale; SDS, Self-Rating Depression Scale.

**Figure 2:**
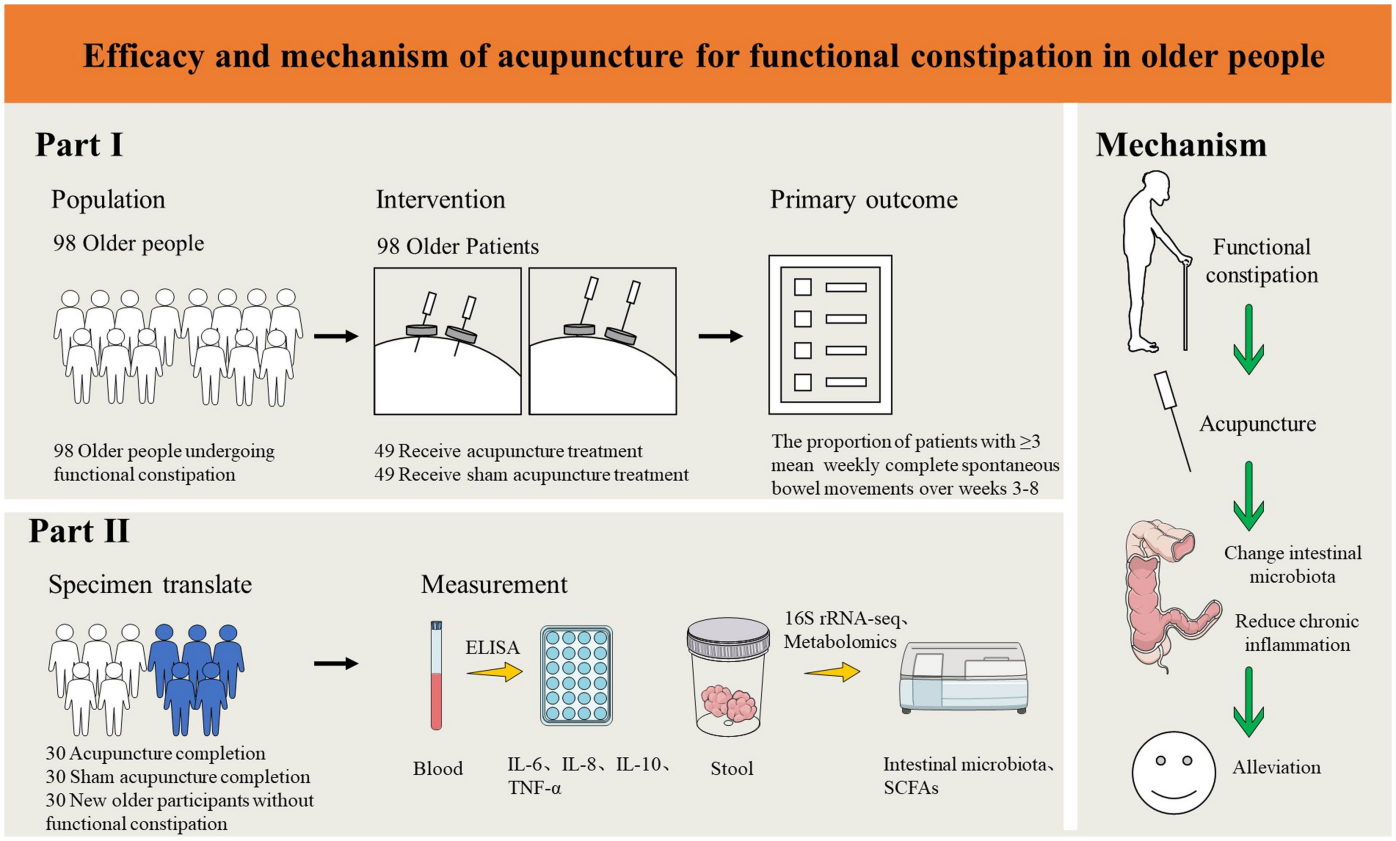
Efficacy and mechanism of acupuncture for functional constipation in the elderly.

This study is a randomized, single-blind, sham acupuncture-controlled trial. It will be conducted at LongHua Hospital Shanghai University of Traditional Chinese Medicine. It has been registered on the China Clinical Trials Registry: ChiCTR2300070735. We will randomly recruit 98 patients who meet the enrollment criteria and randomly assign them to the acupuncture and sham acupuncture groups in a 1:1 ratio. The participants in both groups will be treated for 8 weeks and followed up for 12 weeks. In this clinical trial, we will strictly follow the Consolidated Standards of Reporting Trials (CONSORT) Statement and Standard Protocol Items: Recommendations for Interventional Trials (SPIRIT) (33). A flow chart of the trial procedure is shown in Figure 1 and the efficacy and mechanism diagram of the acupuncture study protocol is shown in Figure 2. Meanwhile, a detailed description of the study procedure and data collection is shown in Table 1.

**Table 1:** Schedule of enrolment, interventions and assessments.

Study period	Enrolment		Intervention period		Follow-up period	
Time point	Week −1	Week 0	Week 4	Week 8	Week 12	Week 20
Eligibility screening	×					
Sign informed consent	×					
Medical history	×					
Randomization		×				
Intervention						
Acupuncture			×	×		
Sham acupuncture			×	×		
Primary outcomes						
Treatment response rate				×		
Secondary outcomes						
Treatment response rate			×		×	×
The percentage of patients with ≥1 increase in mean weekly CSBMs from baseline			×	×	×	×
The mean weekly CSBMs change from the baseline			×	×	×	×
PAC-SYM		×		×		
PAC-QOL		×		×		
SAS		×		×		
SDS		×		×		
Weekly usage of emergency bowel medications		×	×	×	×	×
Adverse events			×	×		
Fecal microbiological		×		×		
Inflammatory cytokines		×		×		
Blinding assessment				×		

### 2.2 Participants recruitment

The recruitment of participants for this study will be done by posting announcements and posters on official web-based information platforms. Interested participants can contact the researcher for initial screening through the phone number provided on the websites. Participants who initially meet the enrollment criteria will then have a face-to-face interview and baseline assessment with an independent researcher. In addition, 30 non-constipated older patients will also be recruited for this trial as a healthy control group who will receive no treatment. All participants will voluntarily sign a written informed consent prior to participation in this study and will be free to withdraw at any time during the study.

### 2.3 Inclusion criteria

For FC patients, the inclusion criteria are as follows:

(1) Meet Rome IV Diagnostic Criteria for Constipation and TCM Diagnostic Criteria of constipation with yin deficiency syndrome; (2) No gender restriction, age 60–80; (3) FC that is classified as mild or moderate (34); (4) Have not used any medicine for constipation at least 4 weeks before treatment (except emergency treatment); (5) Those who can understand various evaluation scales, complete the evaluation and cooperate with the completion of the planned course of treatment; (6) Sign the informed consent.

For healthy subjects, the inclusion criteria are as follows:

(1) No gender restriction, age 60–80; (2) No constipation-related symptoms after comprehensive assessment for enrolment; (3) No significant gastrointestinal symptoms (e.g., abdominal pain, diarrhea, blood in the stool, constipation, abdominal distension, etc.) are reported in the 4 weeks before enrolment; (4) No medication to promote or inhibit gastrointestinal motility within 4 weeks before enrolment; (5) No mental and intellectual abnormalities, able to understand the provisions of each scale and complete the assessment; (6) Agree to participate in the study and sign the written informed consent.

### 2.4 Exclusion criteria

For FC patients, the exclusion criteria are as follows:

(1) Irritable bowel syndrome and constipation caused by tumors, inflammatory reactions, endocrine and metabolic diseases, and drugs; (2) Constipation caused by organic diseases; (3) Patients with serious primary diseases such as heart, brain, liver, kidney and hematopoietic system, infectious diseases, tumors, and mental diseases; (4) Patients with ulcers, abscesses, skin infections, etc. at the acupuncture site; patients with metal allergies or severe fear of needles; (5) Have participated in other medical clinical trials over the past 2 months; (6) Those who used antibiotics or other drugs that may affect gut microbiota and inflammatory cytokines within 4 weeks before enrollment, which may affect the efficacy and judgment; (7) Patients who have received acupuncture treatment within the past 1 year.

For healthy subjects, the exclusion criteria are as follows:

(1) Patients with serious primary diseases such as heart, brain, liver, kidney and hematopoietic system, infectious diseases, tumors, and mental diseases; (2) Have metabolic-related diseases such as irritable bowel syndrome, abnormal thyroid function, or abnormal blood sugar/lipids; (3) Those who have used antibiotics or other drugs that may affect intestinal microbiota and inflammatory cytokines within 4 weeks before enrolment, thus affecting the efficacy and judgment; (4) Participation in other clinical medicine clinical trial studies within the last 2 months; (5) Patients who have received acupuncture treatment within the past 1 month, or will receive acupuncture treatment within the next 2 months.

### 2.5 Randomization and allocation concealment

Participants in the experiment will be divided into two groups by using the method of randomization. Random numbers will be obtained through the “Proc plan” function of SAS 9.4 statistical analysis software by the Clinical Research Center of LongHua Hospital, Shanghai University of Traditional Chinese Medicine. The treatment allocation codes will be placed in sequentially numbered opaque envelopes by an independent study staff member. Depending on the order of the patients’ visits, the investigator will select the envelopes. All participants will be informed that they have an equal chance of being assigned to either the acupuncture group or the sham acupuncture group. Moreover, to ensure the implementation of shielding, all researchers will receive training on the implementation of this research specification many times before the implementation of the experiment and strictly abide by the principle of separation of departments.

### 2.6 Blinding

The single-blind method is used in this trial. With the exception of the acupuncturist, participants, and all study personnel, including statisticians, outcome assessors, sample collectors and data analysts, are unaware of group assignments. To ensure the successful implementation of the blind method, patients are required to wear eye masks during treatment and it is performed in a closed treatment unit. And they will be told not to remove the eye masks during the treatment. In addition, after the last treatment, an independent assessor will provide all participants with three options by asking questions: acupuncture, sham acupuncture, and uncertainty. Then the answers will be recorded on a case report form (CRF). Statistical analysis of patients’ options is performed to assess the success of the blind method implementation.

### 2.7 Intervention

#### 2.7.1 Acupuncture group

Participants in the intervention group will receive acupuncture treatment at Zhao Hai (KI 6), Da Zhong (KI 4), Tai Xi (KI 3), Tian Shu (ST 25), and Shang Ju Xu (ST 37). All acupoints are taken on both sides [Acupoint Location: refer to *The Location of Acupoints: State Standard of the People’s Republic of China* (GB/T 12346–2021)]. All acupoints will be routinely sterilized, and the acupuncture method for each point is shown in Table 2. After the insertion of the needles, manipulations of lifting, twirling, and thrusting are performed on all needles to reach de qi, which is a sensation typically associated with needling including soreness, numbness, swelling, heaviness, and other feelings. This is considered to be an important component of the therapeutic effect of acupuncture (35). The sterile acupuncture needles (size 0.25*40 mm and 0.30*50 mm) that will be used are from Wuxi Jiajian Medical Equipment Co.

**Table 2:** The locations and manipulations of acupoints in the intervention group.

Acupoint	Location	Manipulation
Zhao Hai (KI 6)	On the medial side of the foot, 1 cun below the tip of the medial malleolus, in the depression at the lower edge of the medial malleolus	Insert the needle perpendicularly for 0.5–0.8 cun.
Da Zhong (KI 4)	On the medial side of the foot, posterior and inferior to the medial malleolus, on the upper border of the calcaneus, in the depression on the medial anterior border of the calcaneal tendon attachment	Insert the needle perpendicularly for 0.3–0.5 cun.
Tai Xi (KI 3)	On the posteromedial side of the ankle, in the depression between the tip of the medial malleolus and the calcaneal tendon	Insert the needle perpendicularly for 0.5–1.0 cun.
Tian Shu (ST 25)	On the upper abdomen, horizontal to the umbilicus, 2 cun lateral to the anterior midline	Insert the needle perpendicularly for 1.0–1.5 cun.
Shang Ju Xu (ST 37)	On the outside of the calf, 6 cun below ST 35(Du Bi), on the line connecting ST 35(Du Bi) and ST 41 (Jie Xi)	Insert the needle perpendicularly for 1.0–2.0 cun.

#### 2.7.2 Sham acupuncture group

Participants in the control group will receive sham acupuncture treatment. Sham points, which are 1 cun lateral to Zhao Hai(KI 6), Da Zhong(KI 4), Tai Xi(KI 3), Tian Shu(ST 25), and Shang Ju Xu(ST 37), will be used to match real acupuncture points. The placebo needles chosen for this study are flat-tipped needles without a tip, which could not be pierced into the skin. In the meanwhile, an external patch device will fix the needles, which are visually pierced into the skin. When the blunt tip of the needle touches the skin, patients will feel a pricking sensation, but without the feeling of de qi. After the treatment, the acupuncturist will press the acupuncture point with a dry cotton ball so that the patient can feel the pulling out of the “needle.” It is worth pointing out that the sham acupuncture device used in this study has been granted a utility model patent by China National Intellectual Property Administration (the Patent Number: ZL 2023 20605629.3).

Participants in both groups will be provided with health education, attention to the dietary regimen, avoidance of irritating foods, and attention to emotional self-regulation. Participants will be asked to keep a stool diary in the study, which is recorded during the baseline period (weeks −1 and 0), the treatment period (weeks 3–8), and the follow-up period (weeks 11, 12, 19, and 20). Moreover, the researcher will instruct patients on how to complete the stool diary. All participants will receive 24 sessions of acupuncture or sham acupuncture three times a week for 8 weeks. All treatments will be performed after skin disinfection, and each treatment will last for 30 min. Meanwhile, every patient will be required to stay in the supine position and wear an eye mask during the treatment.

If the patient has had no bowel movement for 3 or more consecutive days, or if the patient has had serious physical discomfort due to the inability to defecate, he/she can take emergency treatment. To be specific, he/she is allowed to use 20 mL of Glycerine Enema (Glycerine Enema, H31021363, Shanghai Yunjia Huangpu Pharmaceutical Co., Ltd.; 20 mL/bottle). However, the patient is required to strictly record the dose and frequency of medication. At the same time, the investigators will record the patient’s medication in detail on the CRF.

### 2.8 Primary outcomes

The primary outcome of the study is the treatment response rate, which is the proportion of participants with ≥3 mean weekly CSBMs over weeks 3–8. The CSBMs refers to the frequency of a bowel movement that occurs in the absence of laxatives or manipulation. In this study, the mean CSBMs at baseline is calculated by dividing the total number of CSBMs between weeks −1 and 0 by the number of weeks 2. Meanwhile, the mean CSBMs at week 8 is calculated by dividing the total number of CSBMs between weeks 3 and 8 by the number of weeks 6.

### 2.9 Secondary outcomes

The secondary outcomes include the following items: (1) The treatment response rate, which is the proportion of participants with ≥3 mean weekly CSBMs during another assessment period. It will be evaluated at weeks 4, 12 and 20. (2) The percentage of patients with ≥1 increase in mean weekly CSBMs from baseline. It will be evaluated at weeks 4, 8, 12 and 20. (3) The mean weekly CSBMs change from the baseline. It will be evaluated at weeks 4, 8, 12 and 20. (4) Patient Assessment of Constipation-Symptoms (PAC-SYM): a scale to assess the condition of the patient’s constipation-related symptoms. It will be evaluated at weeks 0 and 8. (5) Bristol Stool Scale: a scale to classify the patient’s voluntary bowel movement stool. It will be evaluated at week 0 and 8. (6) Patient Assessment of Constipation Quality of Life Questionnaire (PAC-QOL): a brief but comprehensive assessment of the daily life quality of patients with FC, containing 28 items. It will be evaluated at weeks 0 and 8. (7) Self-rating Anxiety Scale (SAS) (36): a measure of somatic symptoms associated with anxiety reactions. It will be evaluated at weeks 0 and 8. (8) Self-rating Depression Scale (SDS) (37): a self-rating scale to assess patients’ depression. It will be evaluated at weeks 0 and 8. (9) Weekly usage of emergency bowel medications: the proportions of participants using medications and doses for emergency treatment. It will be evaluated from week 1 to week 20.

### 2.10 Biological specimen exploratory outcomes

The biological Specimen Analysis Outcomes include the following items: (1) Fecal microbiological outcomes: We will quantify changes in the number and composition of operational taxonomic units (OTUs) of the intestinal microbiota. Changes in intestinal microbiota will be analyzed using 16S rRNA high-throughput detection and the alpha-diversity and beta-diversity will be calculated. Also, high-performance liquid chromatography-mass spectrometry (HPLC-MS) will be used to quantify the amount of intestinal microbiota metabolites Short-chain fatty acids (SCFAs) in fecal. (2) Inflammatory cytokines outcomes: We will use ELISA for serum concentrations of the inflammatory cytokines interleukin-6 (IL-6), interleukin-8 (IL-8), interleukin-10 (IL-10), and tumor necrosis factor-alpha (TNF-α). Fecal and blood samples of the subjects will be collected at week 0 and week 8.

### 2.11 Safety evaluation

Adverse events (AEs) of acupuncture include needle sickness, subcutaneous hematoma, localized infection, dizziness, local pain, aggravation of symptoms and other reactions (38). If any AEs occur, the acupuncturist will promptly assess their severity and take appropriate action. Any AEs occurring during the trial should be filled in the “Adverse Event Form” and further investigated. In the event of any serious AEs, they will be reported to the Safety Committee promptly and dealt with proactively. Meanwhile, a final decision will be made on whether to continue the study. In addition, these AEs data will be evaluated by the investigators in terms of severity and causality to determine whether the AEs are associated with acupuncture or sham acupuncture. The incidence of AEs is expressed as the number of AEs (%) during the study.

### 2.12 Sample size calculation

The sample size is estimated based on the treatment response rate (the proportion of participants with ≥3 mean weekly CSBMs over weeks 3–8). According to the results of our pre-test, the treatment response rate in the treatment group at week 8 was 31.25% (5/16); The treatment response rate of the control group at week 8 was 6.25% (1/16). The test level was set at α = 0.05 (bilateral), and the test efficacy 1-β = 0.80. According to the calculation made by PASS 15.0, the sample size of each group was 39 cases. Considering the 20% shedding rate, 49 cases will be finally taken from each group, and a total of 98 cases will need to be enrolled in this study.

### 2.13 Statistical methods

Statistical analysis will use SPSS 26.0 according to an intention-to-treat analysis. The missing data of the primary outcome will be filled in with the last observation data, while the missing data of safety evaluation will not be filled in. Continuous variables will be expressed as mean ± standard deviation (SD) or median and interquartile range (IQR). The categorical variables will be presented as frequencies or percentages. The *p* value is obtained according to the statistics, and the difference is statistically significant when *p* < 0.05.

Categorical variables, including the treatment response rate (week 8) and the incidence of AEs, will be analyzed using Chi-Square or Fisher’s exact tests. Generalized Estimating Equations (GEE) will be used to compare the data measured repeatedly, including the treatment response rate (week 4, 12, 20), the percentage of patients with ≥1 increase in mean weekly CSBMs from baseline, the mean weekly CSBMs change and Weekly usage of emergency bowel medications. Unpaired *t*-test or Mann–Whitney U test will be used to compare continuous variables between groups, including the mean changes of PAC-SYM, Bristol Stool Scale, PAC-QOL, SAS, and SDS scale.

### 2.14 Quality control, data collection, management, and monitoring

Prior to the start of a clinical trial, all investigators will participate in rigorous training in study protocol, quality control, and data management. The acupuncture therapist for this study must be a licensed acupuncturist with at least 5 years clinical experience, who will receive rigorous standardized training in acupuncture point and non-acupuncture point positioning, acupuncture technique specifications, and how to use the sham acupuncture equipment. Meanwhile, all data collectors will be trained in how to perform data collection, entry, and management prior to clinical trial implementation. CRFs will be used to collect clinical data for each patient, including demographic characteristics, data on individual observables, and AEs. To ensure the accuracy of the data, a dual-entry method will be used. All data will be entered independently by two investigators and then verified to avoid inconsistencies. Once verified as accurate, the data will be entered into the EpiData electronic database for statistical analysis. In addition, a designated clinical supervisor will review the CRF and study the progress regularly.

## 3 Discussion

As a non-pharmacological intervention, acupuncture is a treatment method guided by a holistic concept and has been widely used for FC. Some researchers have confirmed the safety and efficacy of acupuncture in the treatment of FC (39–42). In addition, a meta-analysis, which was based on the comparison between acupuncture and sham acupuncture/medication in treating FC, has concluded that acupuncture is safe and effective in increasing the number of stools, improving stool properties and relieving constipation symptoms, and improving the life quality of patients (43).

Gastrointestinal dysbiosis plays a significant role in the pathogenesis of FC. Some studies have shown that patients with FC have significant differences in their intestinal microbiota compared to healthy individuals (44, 45). Moreover, some studies have found the changing of certain microbial groups in older people (46, 47). Short-chain fatty acids (SCFAs) are the main fermentation products of the gut microbiota (48, 49), which can enhance the motility and contraction of intestinal smooth muscles by reducing the pH value of the gut (50). Additionally, the intestinal microbiota plays an important role in modulating immunity, which can be affected by pathological conditions such as constipation, causing cytokines and chemokines to hyper-secretion (51). A researcher assessed the relationship between inflammatory cytokines and constipation in older adults and found a significant correlation between serum inflammatory cytokines (TNF-α, IL-6, IL-10) and constipation (52–54).

In conclusion, acupuncture has a promising future in the treatment of FC. However, the RCTs specifically evaluating the clinical efficacy and safety of acupuncture in the treatment of FC in older people are lacking, and the mechanism of acupuncture in the treatment of FC is still unclear. Therefore, to explore these issues, we designed the study to assess the effects of acupuncture on FC in older people, with an exploratory look at the effects of acupuncture on the gut microbiota and inflammatory factors. We hope that this study will provide a new understanding of the clinical efficacy and mechanism of action of acupuncture on FC in older people.

However, there are several shortcomings and limitations in this study. Firstly, since this is not a multi-center trial with a large sample size, it may pose a risk that the results cannot be generalized across a wider population. Secondly, due to the nature of acupuncture operations, blinding the acupuncturists is not possible. Thirdly, the study only conducted follow-up observations at 1 and 3 months post-treatment, and no long-term efficacy assessments are conducted. Finally, we only collect blood and stool samples from older patients with FC in this study, but we did not explore the interactions and changes in the central nervous system. These shortcomings will be further improved and compensated in future clinical trials.

## Ethics statement

The studies involving humans were approved by the Medical Ethics Committee of LongHua Hospital Shanghai University of Traditional Chinese Medicine (Ethical approval number: 2023-LHXS-010). The studies were conducted in accordance with the local legislation and institutional requirements. The participants provided their written informed consent to participate in this study.

## Author contributions

YH: Writing – original draft, Methodology. QF: Writing – original draft. YD: Writing – original draft, Methodology, Data curation. XL: Writing – review & editing, Data curation. JH: Writing – review & editing, Data curation. LL: Writing – review & editing, Methodology. YC: Writing – review & editing, Conceptualization. PY: Writing – original draft, Project administration, Methodology.
